# Humanitarian Nationalism During Autocratic Disaster Governance: The Politicization of the Earthquake Response in Türkiye

**DOI:** 10.1007/s12116-024-09454-y

**Published:** 2025-01-24

**Authors:** Cristiana Bellini, Melanie Sauter

**Affiliations:** 1 University of Oslo, Oslo, Norway; 2 University of Mannheim, Mannheim, Germany

**Keywords:** Türkiye, Earthquake, Natural Disaster, Autocratic disaster response

## Abstract

What happens when humanitarian crises are managed by autocratic governments in politicized contexts? This article gives a critical reflection on the 2023 earthquake emergency response in Türkiye. Our study is based on fieldwork interviews and participant observations during the earthquake response. The earthquake shook the country a few months before a contested presidential election. Combining explanations from regime survival theories and disaster policies, we show how elected autocracies strategically contain and co-opt international disaster response mechanisms to reinforce their authority and legitimacy. Yet, international non-governmental organizations (NGOs) can maintain access to such a nationalized response through their financial superiority. We conceptualize the outcome of this nationalized earthquake response as an autocratic aid allocation funnel: a discriminatory aid distribution mechanism favoring the government’s core voter base while marginalizing minorities who lack voting rights. This demonstrates how electoral autocracies use emergencies to strengthen their power. International organizations face a dilemma: whether to provide much-needed aid while potentially becoming complicit in a regime’s unequal and politically motivated disaster response. The case shows how autocratic governments manipulate crises for political gain and exacerbate the vulnerabilities of minorities.

## Introduction

A month after the earthquake, the highway leading to Antakya in the Hatay region is flanked by a haunting landscape of demolished buildings and rugged, pothole-ridden roads. The desolate route is marked by makeshift tents housing displaced individuals, adorned with a diverse array of donor logos, including foreign governments and United Nations (UN) agencies. Yet, amid this mosaic of aid, one emblem remains a constant presence—the Turkish Disaster and Emergency Management Authority (AFAD) logo. This emblem is a symbolic testament to the Turkish government’s pervasive involvement in the earthquake’s emergency response. Upon reaching the UN and non-governmental organizations (NGO) coordination site, Turkish military personnel rigorously inspect all incoming vehicles. This physical interference is metaphorical of the Turkish government’s coercive control over the humanitarian space.

This paper offers insights into how the Turkish state nationalized the humanitarian response of the 2023 earthquake. The study is based on ethnographic fieldwork, including participant observations and interviews with international non-governmental organizations (INGO) staff, as well as an extensive document analysis.

We find that the Turkish government imposed a humanitarian nationalism by assuming a prominent role in the response. It limited the space for INGOs while favoring state-affiliated national NGOs. Because of the presidential elections in May 2023, the government tried to demonstrate full control of the disaster response to increase its voting base. By that it not only constrained the activities of international NGOs but also controlled national organizations through co-optation. The consequence of this humanitarian nationalism was a discriminatory response that particularly disadvantaged the Syrian refugees who could not vote.

Research on the politicization of aid has centered on conflict regions, premised on the notion that armed groups have inherent motives to manipulate aid for political or self-serving ends (Zürcher 2017). Conversely, aid responses to natural disasters in non-conflict zones have traditionally been perceived as “pure” humanitarian crises, where the aid is distributed according to the humanitarian principles of neutrality and impartiality (OCHA 2007). However, our analysis shows that even within well-developed nations, natural disaster relief can become politicized by autocratic regimes. Autocratic governments assert control over civil society, and limit the activities of (international) NGOs (A. Cunningham 2018; Dupuy et al. 2016; Heurlin 2010). This once again presents aid agencies with the challenge of how they can stay neutral and impartial when increasingly autocratic governments stand in their way.

Our findings have implications for both governance and humanitarianism. When autocratic governments manage crises, political agendas compromise humanitarian priorities, leading to biased aid distribution. This discrimination against marginalized groups also contributes to the erosion of democratic processes. Understanding how autocratic regimes deal with crises sheds light on power dynamics, state-society relationships, and the effectiveness of humanitarian interventions. The case of the 2023 Türkiye earthquake highlights the intersection of a natural disaster with a politically charged environment, and how autocratic governments exploit crises for political gain and exacerbate the vulnerabilities of minorities.

This topic intersects with existing literature on disaster governance (Hilhorst et al. 2020), authoritarianism (A. J. Cunningham 2023), and humanitarianism (del Valle & Healy 2013). Scholars have examined how autocratic regimes exploit disasters to consolidate their power (Flores & Smith 2013), maintain control (Wood & Wright 2016), and shape public narratives (Windsor et al. 2014). Drawing on regime survival theories and disaster politics, our research contributes to understanding the mechanisms through which electoral autocracies exploit crises to strengthen their rule.

This article is structured as follows. “Natural Disasters in Authoritarian States” provides a discussion on how natural disasters affect authoritarian states and the role of humanitarian aid. In “Research Design,” we explain our research design based on fieldwork. “Analysis: National Control over the Humanitarian Space” presents the main analysis, showing how the Turkish government nationalized the humanitarian space. We give concluding remarks in “Conclusion.”

## Natural Disasters in Authoritarian States

Most humanitarian emergencies do not take place in model democracies. A natural disaster becomes a humanitarian emergency based on political failures before the actual natural event. 68% of the world’s population now resides in autocracies (Quraishi 2022). Türkiye under Erdogan’s rule has been one of the most prominent example of an electoral autocracy (Mechkova et al. 2017).

The politicization of humanitarian aid is a well-documented phenomenon that occurs across political regimes, from democracies to non-electoral autocracies (Duffield et al. 2001; Lischer 2006; Sauter 2023). In democracies, politicization tends to manifest through the strategic distribution of aid to favor political constituencies, especially in the lead-up to elections. Transparency mechanisms and electoral competition generally curtail outright capture (Bommer et al. 2022; Jablonski 2014). In contrast, non-electoral autocracies use aid to consolidate power, often prioritizing loyalty and regime survival over impartial distribution (Kono & Montinola 2013; Paik 2011). Electoral autocracies like Türkiye sit at the intersection of these two dynamics, where governments face electoral pressures but also have the tools of authoritarian control at their disposal. In such regimes, the politicization of aid serves dual purposes: bolstering the regime’s legitimacy among voters and co-opting civil society organizations to prevent them from becoming vehicles of dissent (Ertas 2024; Gerschewski 2013).

During natural disasters, such as famines, more people starve in autocracies, showing the need for robust institutions to mitigate disaster-related fatalities that are “man-made” (Sen 1983). The number of fatalities during natural disasters in autocratic regimes does not significantly impact protest movements against or the survival of the government. In democracies, ineffective disaster response jeopardizes politicians’ chances of re-election. However, the mere incidence of a natural disaster can destabilize autocratic leadership, unlike in democracies (Flores & Smith 2013). In autocracies, governments maintain power by appeasing their coalition cronies with private goods and access to government resources. Natural disasters increase the chances of government replacement (Chang & Berdiev 2015).

Natural disasters impose significant costs on vulnerable states and can strain state-society relations, altering domestic political dynamics. Disasters reshape state authority and introduce new actors, potentially influencing dissent and repression within affected states. Grievances increase after natural disasters and the state loses control, which creates opportunities for dissent and challenges to authority. In the aftermath of natural disasters, autocratic leaders, feeling threatened in their survival, frequently resort to violent repression against their population (Wood & Wright 2016). If economic inequality and political dissent are already pervasive before the disaster, autocratic governments tend to react even more harshly to the shock (Pfaff 2020). Post-disaster humanitarian aid mitigates the impact of disasters on repression, especially in more democratic states (Wood & Wright 2016). Autocrats strategically use blaming and credit-claiming language in the aftermath of natural disasters to prolong their time in power. Effective disaster responses can bolster the leader’s authority, while ineffective ones may weaken it (Windsor et al. 2014).

### Humanitarian Disaster Response in Autocracies

A disaster is viewed as an apolitical humanitarian emergency because, in principle, the disaster itself is not subject to political interests, unlike humanitarian emergencies in conflict regions. At the same time, government control of the response and a lack of principled action appear less concerning than in violent conflicts (OCHA 2007).

The increasing global autocratization is a problem for the politicization of a government-controlled emergency response. Authoritarian governments are sceptic towards humanitarians. The mere presence of humanitarian actors is perceived as a threat to their authority (A. Cunningham 2018; Schenkenberg 2016). NGOs can serve as a check against the expansion of state power, particularly when they maintain independence from government influence (Müller 2006; Shils 1991).

However, the state can also exert control over NGOs, shaping their agendas to align with its ideological objectives. This dynamic can lead to civil society becoming an instrument of hegemonic forces, spreading the ideology of the ruling class and reinforcing its power through nonviolent means (Katz 2010). NGOs become increasingly entangled with governments in authoritarian contexts. They are not only instrumentalized by authoritarian governments for legitimization, but also subject to repression, and co-optation, and are targets of politicization efforts to align them with regime interests (Gerschewski 2013). In some cases, governments seek to involve NGOs in service provision to enhance their legitimacy and stabilize the regime (Lorch & Bunk 2017).

Authoritarian governments tend to develop a “humanitarian nationalism” where humanitarian action is highly controlled and nationalized through the marginalization of NGOs and replaced by state institutions (Churruca-Muguruza 2018; Heurlin 2010). Autocratic states use NGOs as tools to maintain their authority. Financial and administrative links between NGOs and the state limit their ability to challenge government policies or actions. In Türkiye, the state has historically oscillated between restricting, controlling, and sometimes supporting NGOs based on their alignment with state interests. Different political contexts have led to varying degrees of tolerance or repression towards certain types of NGOs, with religious, Kurdish, leftist, and politically divided organizations facing scrutiny and closure at different points in Turkish history (Göle 1995).

Authoritarian regimes use various strategies to prevent societal organizations from engaging in mobilized dissent, including repression, coercion, co-optation, and containment. Autocratic governments repress claims-making NGOs with ideological agendas but support loyal NGOs and accept service-providing NGOs (Toepler et al. 2020). Securing access to operate in authoritarian environments hinges on demonstrating the relevance of their services and building relationships with authorities and potential allies within ministries. Organizations need to prove their added value while navigating compromises against ethical principles (del Valle & Healy 2013).

Regimes respond to perceived NGO threats with registration requirements, the creation of state-controlled entities, fundraising restrictions, and direct harassment (Heurlin 2010). Co-optation involves integrating organizations into state institutions to ensure loyalty to the regime. Organizations in cohesive networks with compatible interests are typically co-opted because they possess significant mobilization potential, and autocrats aim to ensure their interests align with the regime. Containment refers to the conditional toleration of groups outside state institutions, aiming to neutralize their potential threats. Organizations in loose networks with reconcilable interests are contained to prevent them from challenging leadership, despite their lower potential for large-scale militancy (Reny 2021). The Turkish Red Crescent is an example of a co-opted organization.

Under the corporatist approach, the state integrates NGOs into its framework by imposing stringent registration procedures, establishing government-controlled NGOs (GONGOs) to preempt independent initiatives, and co-opting NGO leaders into state structures (Foster 2001; Lehmbruch & Schmitter 1982). The exclusion strategy aims to hinder NGO growth by subjecting them to harassment, restricting their operational scope, and replacing their functions with state-run institutions (Bratton 1989; Foster 2001). The choice between violent and administrative crackdowns on NGOs hinges on two factors: the immediacy of the threat they pose and the consequences of repression. Violent tactics are favored for immediate threats like protests but risk backlash, reduced legitimacy, and human rights violations. Administrative measures are preferred for long-term strategies, especially when NGOs could influence elections or challenge state interests. They carry fewer domestic and international consequences, being perceived as regulatory rather than repressive actions (Chaudhry 2022).

Some nonprofits opt to maintain neutrality to ensure continuity and security of resources, prioritizing service provision over political engagement. This apolitical service provision is welcomed even in autocratic regimes (Aasland et al. 2020; Salamon & Toepler 2015). However, their proximity to authoritarian regimes may lead to co-option or manipulation by the state, potentially undermining their independence and societal impact. While loyal NGOs align themselves with the ideologies of the regime, neutral NGOs are co-opted or politicized, leading to a blurring of lines between civil society and the state. At the same time, service-only providing NGOs are not opposition forces that need to be co-opted. The regime utilizes them to bolster its voter base while simultaneously depoliticizing service provision (Toepler et al. 2020).

External disaster aid poses challenges for autocratic regimes. Since disasters imperil the survival of autocracies, such governments often attempt to control the narrative by suppressing or altering information post-disaster (Stasavage 2020). The dispersal of an international aid response can make it more difficult to keep the narrative and behavior of all aid actors under control.

Furthermore, aid has direct and indirect mechanisms that affect people’s behavior. On the one hand, it can positively influence government performance, leading to positive perceptions of the government. On the other hand, it can also show that the government alone cannot manage the emergency. When international aid organizations intervene, it signals to the population that the government cannot handle the crisis alone. If essential services are provided by foreign aid, it can impact people’s perceptions of government effectiveness and legitimacy (Chang & Berdiev 2015). Consequently, autocratic governments often resist outside disaster aid and carefully evaluate needs and risks before accepting international assistance (Paik 2011).

International aid affects domestic governance processes in recipient countries in different ways, dependent on the assistance provided (aid level), the influence exerted by aid donors in determining the aid design, and the methods through which aid is administered. Facilitative aid dynamics are characterized by significant international support for government-driven agendas, enhancing state effectiveness by supplementing government efforts without overshadowing their autonomy. Conversely, directive aid dynamics involve heavy donor influence, potentially compromising government priorities and autonomy in service delivery (Barma et al. 2020). Facilitative aid can enhance state legitimacy by supporting indigenous efforts, improving service delivery, and building government capacity. It suggests that effective aid can contribute to both tangible service provision and symbolic reform objectives, ultimately bolstering government legitimacy and state-society relations (R. A. Blair & Winters 2020; Schmelzle & Stollenwerk 2018). The basic assumption is that this is simply positive. But in autocracies where the government enforces this model, it can lead to biased, unprincipled, and inefficient aid delivery.

Regime survival theories help explain why autocratic regimes often politicize humanitarian aid during crises. In non-electoral autocracies, the absence of public accountability allows leaders to focus on maintaining elite loyalty through selective distribution of resources (Flores & Smith 2013). However, in electoral autocracies, such as Türkiye, the need to win elections forces regimes to balance elite interests with mass appeal. Disasters introduce additional volatility, as the regime must appear responsive to the needs of its population while maintaining control over civil society organizations (Wood & Wright 2016).

This paper contributes to this growing body of literature by examining the mechanisms through which electoral autocracies manage humanitarian crises, using Türkiye’s 2023 earthquake response as a case study. Administrative structures within organizations can only be co-opted if they operate at a national or regional level. International organizations are predominantly subjected to administrative constraints that hinder their operations. In this article, we build on these insights to show how the political dynamics of the upcoming elections coupled with the exogenous shock of the earthquake threatened Erdogan’s regime. To protect his political interest, Erdogan claimed credit for the earthquake response by co-opting national NGOs and containing INGOs. This humanitarian nationalism resulted in a discriminatory aid response.

### The INGO Purge in 2017

Since 2011, Türkiye has become a major host country for refugees escaping the Syrian civil war, sheltering 3.6 million registered Syrians (UNHCR 2023).

Gaziantep, the nearest major city to the Syrian border, is a hub for international NGOs assisting the refugees. AFAD took charge of refugee coordination with the 2014 Regulation on Temporary Protection for Syrian Refugees. The government agency controls refugee camp management and access for independent observers, journalists, NGOs, and relief organizations (Memisoglu & Ilgit 2017).

Initially, many local and international organizations were involved in the refugee response. However, after the failed coup attempt against President Erdoğan in 2016, the government orchestrated a massive purge of dissidents (Mellen & Lynch 2017). The state took control over INGO operations, at times banning them from operating in Türkiye. Several US-based NGOs were targeted because of the US support for the Syrian-Kurdish operations in Syria. The Turkish government believed that these NGOs were assisting the Kurds in Türkiye through cross-border operations (Longton 2017).

Furthermore, the state revoked the permits of several international organizations, resulting in a “purge” of INGOs by the Turkish government, along with the detention and deportation of Syrian aid workers (Longton 2017). Subsequently, bureaucratic hurdles were introduced, requiring NGOs to hire more Turkish nationals and provide extensive documentation of funding and aid recipients. Work permits were issued slowly and arbitrarily. In 2017, the tensions between foreign NGOs and local authorities were so high that the police would frequently make incursions into restaurants frequented by foreign aid staff to check work permits (Boztaş, 2019). Many analysts interpreted these moves as Erdoğan’s attempt to consolidate power, targeting organizations suspected of collaborating with groups opposed to the Turkish government (Mellen & Lynch 2017).

This suspicion of outsiders and the narrative of NGOs conducting clandestine operations led to arbitrary measures, including police inspections in INGO offices and popular cafes of their staff, detention and deportation of foreign aid workers, suspension or review of operating licenses, and the implementation of restrictive protocols. By imposing institutional conditions that hindered aid flows, the Turkish government played a critical role in shrinking the humanitarian space. State-affiliated domestic organizations replaced international NGOs (Boztaş, 2019).

Even before the earthquake, Türkiye was grappling with a humanitarian crisis due to the influx of Syrian refugees. The humanitarian space had already been heavily politicized. Erdogan was concerned that NGOs might align with his political rivals; therefore, he controlled national organizations and expelled INGOs from the country or severely limited their operations. This illustrates how, even before the earthquake, INGOs faced restrictions or expulsion, while only national organizations that could be co-opted by the government were allowed to operate. Erdogan viewed INGOs, especially rights-based ones, as a threat to his regime and political survival, fearing they could rile up civil society. In our analysis, we show how this fear peaked with the exogenous shock of the earthquake, which hampered his political popularity ahead of the upcoming elections. Paradoxically, he could not simply throw out the international organizations anymore because his political survival depended on their resources to make the response more efficient.

To further contextualize the Turkish case, it is useful to compare it to other disaster responses in autocratic settings. For instance, in China’s 2008 Sichuan earthquake, the government allowed a relatively open international response initially. However, as the immediate crisis subsided, the Chinese government began to restrict the role of international actors and clamp down on independent reporting (Paik 2011). This gradual restriction reflects the Chinese regime’s strategy of maintaining strict control over civil society and narratives, especially as public criticism about corruption in the construction sector began to emerge (Stasavage 2020). Unlike Türkiye’s electoral autocracy, where Erdogan had to balance the need for international aid with his electoral ambitions, China’s non-electoral regime faced no such pressures. Instead, its approach focused entirely on suppressing any dissent that might threaten the regime’s legitimacy. In this context, aid politicization took the form of strict state control over the dissemination of information and resources, emphasizing regime stability over public accountability. This contrast with Türkiye highlights how electoral pressures force regimes like Erdogan’s to maintain a more delicate balance between controlling aid and engaging with international actors to project competence.

Similarly, Myanmar’s military junta, another non-electoral autocracy, exhibited a rigid approach to international humanitarian intervention following Cyclone Nargis in 2008. The government initially refused to allow international aid workers into the country and was criticized globally for delaying aid distribution to over 2 million affected people (Paik 2011). Here, the regime’s fear of external influence and loss of sovereignty over domestic affairs took precedence, leading to a strategy of isolation rather than co-optation. Myanmar’s case shows how non-electoral autocracies tend to resist external aid to protect regime sovereignty, an approach that differs markedly from Türkiye, where international organizations were allowed entry but had to conform to government-dictated terms. The Turkish government needed the resources and operational expertise of international actors but sought to claim credit for the aid provided, particularly in the lead-up to the 2023 elections.

In contrast, the response to the 2010 earthquake in Haiti, and the 2015 earthquake in Nepal, both (fragile) democratic regimes, exhibited different patterns of aid politicization. In Haiti, the weakness of the state led to the overwhelming dominance of INGOs and international organizations, which effectively sidelined the government in the relief effort (Schuller 2016). This resulted in a form of “reverse politicization” where the lack of state control led to disorganized and inefficient aid distribution, ultimately undermining the Haitian government’s legitimacy. Similarly, Nepal’s response to the 2015 earthquake was marked by intense competition between political parties to control aid distribution, with the central government struggling to coordinate efforts among various international and local actors (Lee 2016). Unlike Türkiye, where the central government tightly controlled the narrative and the aid process, democratic regimes like Haiti and Nepal struggled with fragmented responses due to the competing interests of various political factions and international actors. This difference underscores how democracies, particularly fragile ones, are more likely to experience decentralization or competition over aid control, as opposed to the centralization observed in electoral autocracies like Türkiye.

These examples show the central role that regime type plays in shaping the politicization of aid during disasters, linking directly to theories of regime survival and disaster politics. In non-electoral autocracies like China and Myanmar, the absence of electoral competition allows regimes to focus on elite consolidation and sovereignty preservation, often at the expense of effective aid distribution. These regimes tend to view external humanitarian actors as threats to state sovereignty and seek to exclude or tightly regulate them to prevent challenges to their authority (Paik 2011; Stasavage 2020). In contrast, electoral autocracies like Türkiye must navigate the dual pressures of maintaining elite support while also appealing to the electorate. As Flores and Smith (2013) argue, disasters in such regimes pose unique threats to leader survival because the regime must appear responsive while maintaining control over both domestic and international actors.

Furthermore, in fragile democracies like Haiti and Nepal, the politicization of aid is more fragmented, with political factions competing for control over resources, often leading to inefficient or corrupt distribution mechanisms. These cases align with Schmelzle and Stollenwerk’s (2018) argument that in areas of limited statehood, the state’s inability to control the narrative or the distribution of aid weakens its legitimacy and governance capacity.

## Research Design

This qualitative case study relies on fieldwork, including participant observation and semi-structured interviews, as well as an extensive document analysis of situation reports from humanitarian organizations involved in the earthquake response.^1^

One of the authors was initially conducting fieldwork in Gaziantep, a city in Southern Türkiye, for a separate study when a 7.8-magnitude earthquake struck Gaziantep on February 6, 2023. Nine hours later, a 7.5-magnitude earthquake hit Kahramanmaraş. The earthquakes heavily impacted Northern Syria too. The Turkish Government declared a 3-month state of emergency in the hardest-hit areas including Gaziantep, Kahramanmaraş, Hatay, Adıyaman, Diyarbakır, Adana, Osmaniye, Şanlıurfa, Malatya, and Kilis (OCHA 2023a). In total, more than 50,000 people lost their life (OCHA 2023b). The earthquake’s material damage is estimated to exceed $100 billion USD. 214,577 buildings are heavily damaged, demolished, or in urgent need of demolition.

The author was affiliated with an INGO involved in the Syrian refugee response. Following the earthquake, the author volunteered with the INGO’s emergency response for 2 months. Throughout this period, the author consistently disclosed their researcher status to all INGO staff they engaged with, and both the staff and the organization provided their consent for the use of the observations made during the volunteering.^2^

During this time, the author not only interviewed INGO staff but also participated in meetings and field activities such as aid distribution. This immersive experience within the organization provided exclusive access to observe NGO staff in various social contexts, both within and beyond their official working hours. This allowed them to understand the organization’s internal dynamics, as they learned their codes and values, which were essential for comprehending the meaning behind their actions (Silverman 2013).

The team of experts that we will call the emergency team was deployed right after the earthquake and counted 13 expatriates from various countries, each bringing a wealth of experience and specialized knowledge to the crisis response. This study is based on the author’s observation during their 2 months volunteering period and constant interactions and conversation with the emergency team. These observations are further enriched by six formal interviews conducted with the INGO staff 2 months after the earthquake took place. The interviewees include one representative from the country office and five members of the emergency team. The interviewees represent a diverse group of mid- and senior-level international staff who were deployed because of their expertise in critical humanitarian sectors, including logistics, shelter, protection, and disaster management. Each of these individuals has a robust background, having been deployed in multiple emergency operations across the globe. However, none of the emergency team members had previous working experience in Türkiye. Expats from different countries may carry inherent biases based on their cultural and national backgrounds. For instance, those from countries with strained relations with Türkiye may have a predisposed scepticism of the government. By examining their perspectives, this study aims to illuminate shared sentiments among the interviewees, as well as any areas of disagreement, thereby providing a comprehensive analysis of the dynamics at play during the emergency.

We triangulate our information with weekly situation reports from different UN agencies, the IFRC, the Turkish Red Crescent, government announcements, and social media monitors (see Appendix). Combining multiple data collection techniques, including participant observation, interviews, and document analysis allows for verification of the information, enhancing the overall reliability and validity of the study (Kapiszewski et al. 2015).

The affiliation with the organization and the personal relation with the interviewees might add potential biases to the analysis as it undermined the researcher’s independence and objectivity. To minimize this bias, a second author that was not present in the earthquake response helped analyze the material.

## Analysis: National Control over the Humanitarian Space

**Fig. 1:**
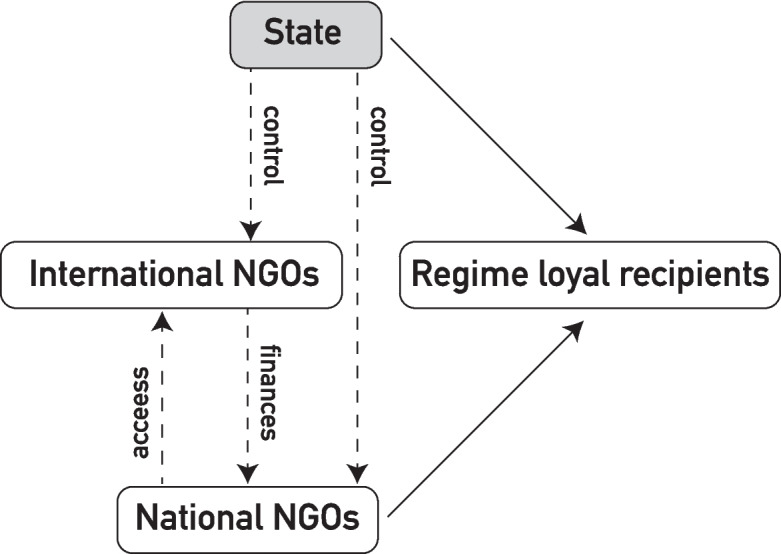
The aid allocation funnel of an electoral autocracy

We show how the Turkish government nationalized the earthquake response for political aims, and how this had dire effects for minorities without voting rights. Figure 1 illustrates our concept of the aid allocation funnel in an electoral autocracy. The Turkish government’s co-optation strategy created a group of state-affiliated organizations which extended the state’s influence into the humanitarian space. This is extraordinary because in most other humanitarian emergencies, INGOs control national NGOs. In the Turkish case, the State controls national NGOs and INGOs, and forces INGOs to operate through state affiliated NGOs.

INGOs can influence local partners through financial resource, thereby getting access through a national NGO. Simultaneously, national organizations are dependent on INGOs due to their financial dominance. Even the state relied on INGOs, allowing them to enter and support the response, albeit under state-imposed conditions. The government-controlled response ultimately resulted in a nationalist humanitarianism, where the government focused its efforts to regime loyal recipients.

The politicization of the earthquake response can be understood through the lens of *regime survival theories*. Erdogan’s government faced significant electoral pressure, as the earthquake struck just months before the presidential elections. In electoral autocracies like Türkiye, crises such as natural disasters represent both a threat and an opportunity for political leadership (Flores & Smith 2013). On the one hand, a poorly managed response could further erode public trust, which was already declining due to economic instability and inflation. On the other hand, a tightly controlled, nationalist response presented an opportunity for Erdogan to consolidate support among core constituencies, demonstrating his regime’s competence and leadership. This need to preserve electoral dominance drove Erdogan to centralize the aid distribution process through state-affiliated organizations like AFAD and the Turkish Red Crescent. By doing so, the regime was able to project an image of control, downplay the role of INGOs, and selectively channel aid toward loyal voter bases while marginalizing vulnerable groups like Syrian refugees who lacked voting rights. This pattern aligns with broader disaster politics theories of autocracies, which posits that authoritarian leaders use crises to bolster their political survival by controlling the narrative and delivery of humanitarian aid (Windsor et al. 2014). The Turkish government’s manipulation of aid distribution during the earthquake highlights how electoral considerations fundamentally shaped the response, creating a feedback loop where the regime’s survival depended on the politicization of humanitarian resources.

### The Earthquake and the Presidential Election

The earthquake in Türkiye struck at a politically delicate moment. Three months later, the country was gearing up for presidential elections where Erdogan encountered a formidable and united opposition candidate for the first time. Türkiye was grappling with severe inflation exacerbated by the aftermath of COVID and the Ukrainian conflict. The country faced an economic crisis with a 58% inflation rate registered in January 2023 (Reuters 2023).

Due to the challenging economic conditions, public support for the government waned. Against this backdrop, taking control of the earthquake response became critical. A caravan of international NGOs participated in the earthquake response, providing neutral service provisions. However, these efforts were politicized and co-opted by the regime. We demonstrate Erdogan’s strategic manipulation of the earthquake response to bolster his voter base, portraying it as solely the government’s endeavor in the lead-up to the presidential election. This resulted in an inefficient response that prioritized political gains over addressing genuine needs.

Many blame Erdogan’s desire for power and the associated inefficient centralization of state institutions for the inefficient earthquake response. He has undermined and weakened important institutions and appointed loyalists who do not have the necessary qualifications to key positions, a prime example of state co-optation (Luhn 2023). The leading role of the government restricted the activities of national NGOs and INGOs and hampered an efficient distribution of aid. The response was marked by political motivations influencing aid distribution, with reports suggesting favoritism towards those with similar political affiliations, leading to uneven targeting of aid. Lack of systematic needs assessments allowed political biases to flourish (F. Blair 2023).

The response to the earthquake was led by the Turkish government and coordinated through AFAD. This political control was essential for Erdogan’s political survival, yet it also proved to be a liability when circumstances turned unfavorable. The government was criticized for the slow response (Browne 2023). Moreover, despite existing earthquake regulations, widespread corruption among local and national authorities had allowed construction projects that did not adhere to anti-seismic rules, contributing to the extensive destruction caused by the earthquake (Yeginsu et al. 2023).

The corruption scandals related to earthquake protection and building regulations further tarnished the image of the Erdogan government (Hattam 2023). Many prominent news outlets worldwide covered how the corruption exacerbated the impact of the earthquake (Links 2023; Yeginsu et al. 2023). Three days after the earthquake, public criticism of the government was so high that the government blocked access to Twitter in the whole country (Links 2023). The social media shutdown underscores the government’s priority to control the narrative of the response, leading to the suppression of free information. Prosecutors opened investigations into journalists and social media users who criticized the earthquake response (CPJ 2023). This repressive tactic demonstrates that the regime perceived criticism of the response as an immediate threat.

Social media discussions related to the aid sector tended to gravitate heavily around the contested response to the crisis of the Turkish Red Crescent (TRC) (Insecurity Insight 2023b). The TRC leadership was accused of personally profiting from the sale of tents (medya, 2023). This situation also posed challenges for Erdogan and his government. Since the TRC was closely associated with the state, the corruption scandal was linked to the government. Just a few weeks before the elections, Erdogan found himself compelled to publicly condemn the TRC to distance himself from the scandal. Shortly thereafter, the head of the TRC had to step down from their position (Insecurity Insight 2023a).

**Fig. 2:**
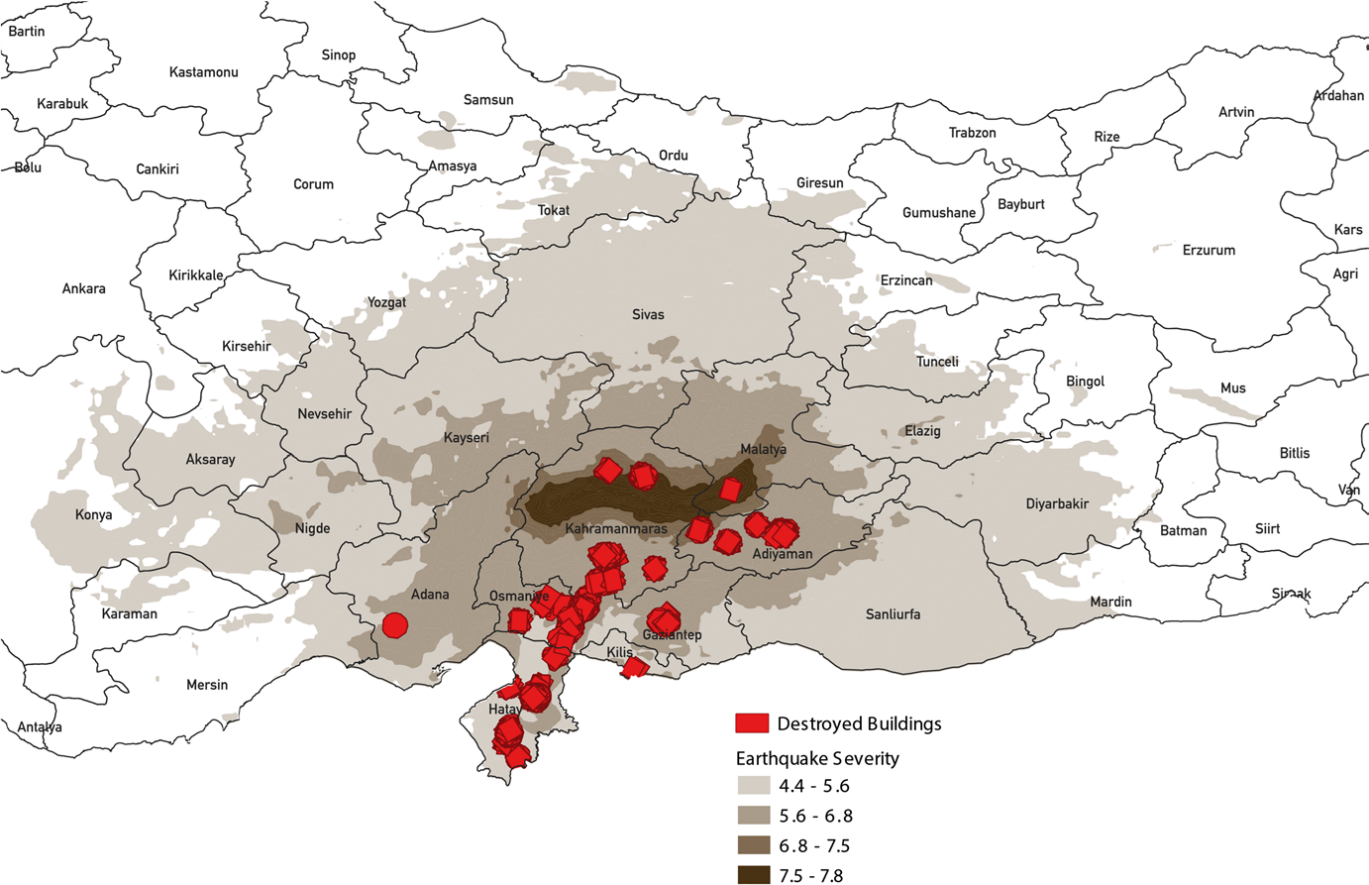
Earthquake affected locations and damaged building Source: Data from ShakeMap / United States Geological Survey (USGS) and OpenStreetMap

**Fig. 3:**
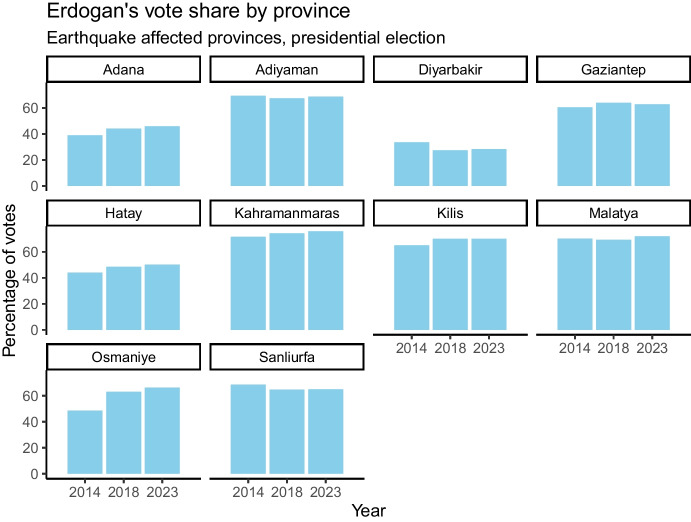
Election data from the presidential elections in Türkiye Source: Data from ‘turkeyelections’ R package by Ozancan Ozdemir (2024)

Figure 2 shows the earthquake affected regions and the severity of the earthquake, and the areas with most destructed buildings (red polygons). The map shows that Adiyaman, Gaziantep, Hatay, and Kahramanmaraş were most affected by the destruction of the earthquake. Figure 3 shows the vote share for Edogan in the earthquake affected regions for the 2014, 2018, and 2023 presidential elections. Besides Hatay, the most affected provinces were also of core importance for Erdogan in the election, as in these regions he had a majority of voters. Consequently, it became imperative for the government to be perceived as effectively addressing the earthquake and its aftermath to regain public trust.

### Government Control of Earthquake Response

AFAD has been the most prominent governmental body in the earthquake response, managing the national coordination of the emergency response, and more specifically the provision of tents in formal settlements.^3^ The disaster response control from the Turkish government is highly centralized, even the municipalities needed permission from the national government to implement activities.^4^

The Turkish government collaborates with national NGOs that share its policies, creating a group of “favoured, state-affiliated NGOs” (Boztaş, 2019). In the earthquake emergency response, the most important local organization has been the TRC. TRC is the national partner of the International Federation of Red Cross and Red Crescent Societies (IFRC). TRC has also been the largest national recipient of international funding (Girling-Morris & Bahçecik 2022). Even though the TRC claims to be a neutral humanitarian organization, it is considered a state-affiliated organization (Boztaş, 2019), some calling it a Turkish official institution at the same level as AFAD (Aras & Duman 2019). In fact, the IFRC has criticized the TRC in the past about its lack of impartiality and independence from the state (Paker 2007). One interviewee argued that both in Türkiye and in Syria, the Red Crescent societies are co-opted by the government.^5^

Data from the UN’s Türkiye Earthquake Flash Appeal 2023 highlights a significant bias in favor of international organizations and NGOs over national and local entities in terms of direct international funding.^6^ Seventy-four organizations participated in the UN coordination mechanism.^7^ The government faced the challenge of regulating and asserting ownership over the activities of INGOs without exacerbating an already ineffective response. In other words, the government had to find a way to achieve a facilitative earthquake response with significant international support for its own agenda, and where the resources of INGOs enhanced state effectiveness by supplementing government efforts without overshadowing their autonomy (Barma et al. 2020). The dilemma was that the regime perceived the already present organizations capable of rapidly expanding earthquake relief efforts as political entities primarily focused on aiding Syrian refugees, rather than impartial service-oriented organizations.

The government nationalized the humanitarian space. It not only introduced laws to impose a ratio between Turkish and expat nationals working for NGOs but it also pushed the INGOs to partner with Turkish organizations to keep their activities in the country.^8^ Türkiye has a history of a nationalized humanitarian space. Already after the earthquake in 1999, the government relied heavily on “state-friendly” NGOs (Jalali 2002). During the INGO purge of 2017, many foreign NGOs have been expelled because they would not collaborate with the local authorities and organizations.^9^

The emergency team perceived that the government uses INGOs either as implementers serving the government or to use the INGOs funding for their purposes.^10^ For example, in shelter camps for the displaced from the earthquake, all the tents, regardless of the organization that procured them like UNHCR or USAID, had the logo of AFAD. This created the perception that it was indeed the government managing the response. Four interviewees pointed out how the Turkish government tried to assert its control over the humanitarian space.

“*In Türkiye, there are multiple levels of complexities and some of them have to do with the level of control that the Turkish government is willing and intends to have over the situation. So, I would say that the Turkish government did not allow a lot of space for humanitarian organizations to step in and provide support, even though the needs are quite pressing. There are limitations in terms of access, so outreach, for instance, is quite restricted.*”^11^

AFAD did not want the INGOs to distribute tents because it wanted to demonstrate its leading role in the emergency, but the demand turned out to be so high that AFAD and the TRC could not tackle it. Eventually, INGOs supported tents, but so late that distribution for people in need was severely delayed. Furthermore, one interviewee recalled how the INGOs were limited to the supply of the tents but not having a say in their distribution:

“*…[local authorities] were asking the [INGO] to provide support. But not provide support like, go there and do your activity. Provide support like, buy tents and give them to us*”*.*^12^

**Fig. 4:**
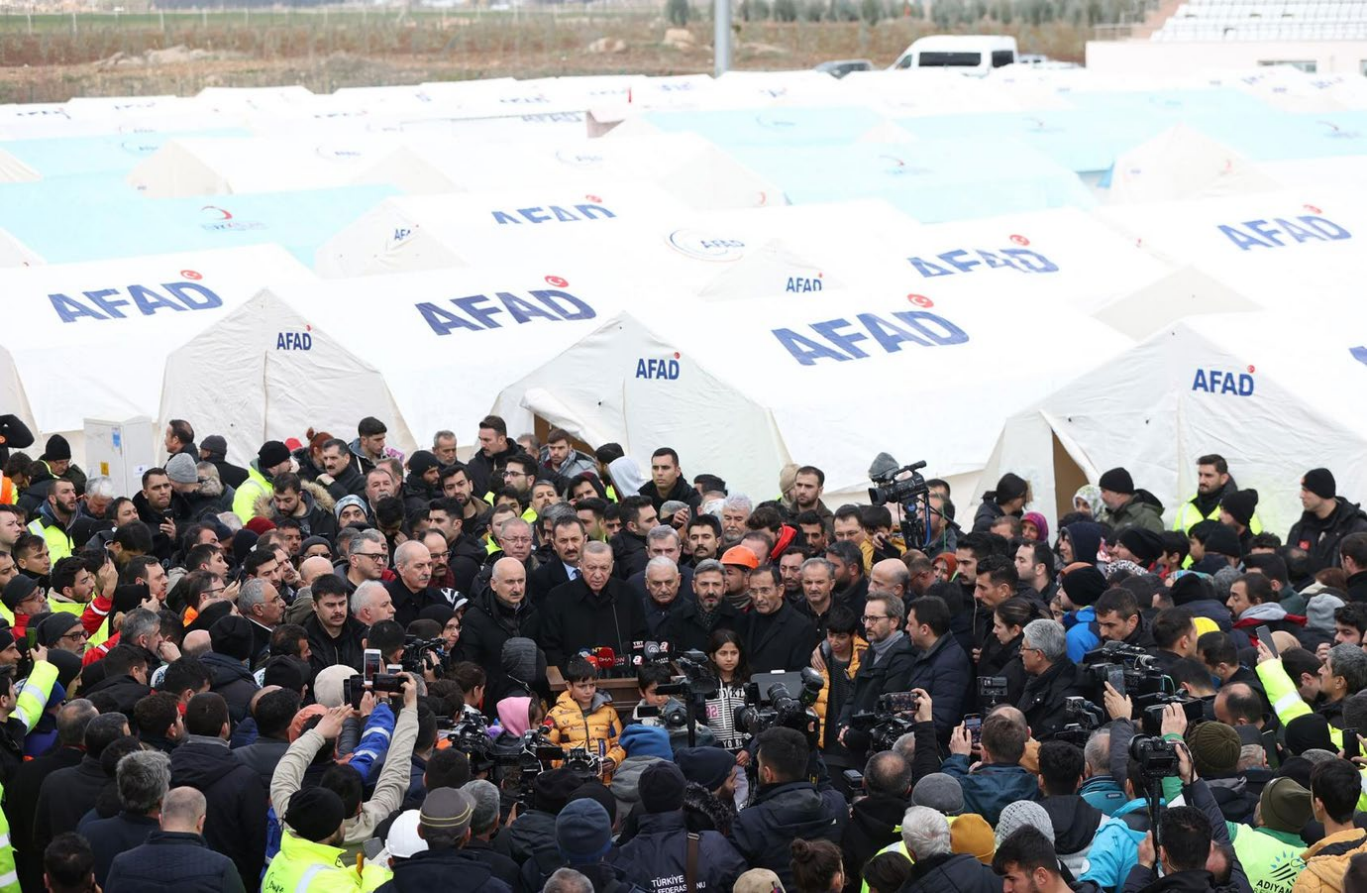
Erdogan speaks to media in Adiyaman Source: Murat Kula—Anadolu Agency/Getty Images, 10.02.2023

In the beginning of this article, we highlighted the conspicuous presence of AFAD logos. This was a deliberate signal to the population aimed at projecting an image of being in control of the earthquake situation.^13^ Figure 4 shows Erdogan in a temporary camp for earthquake victims holding a speech in front of a large gathering of men. In the background, all tents carry the AFAD logo. The picture illustrates Erdogan leveraging the situation to gain political advantage.

Erdogan’s regime, already weakened by economic crises and public dissatisfaction, viewed the earthquake as a potential threat to its survival. As a result, the Turkish government sought to monopolize the narrative surrounding the disaster response, co-opting INGOs and directing aid toward its core constituencies to consolidate electoral support (Blair 2023). This is consistent with the broader literature on disaster politics, which suggests that autocratic regimes use crises to project strength, but the need for external resources often complicates their ability to fully control the aid apparatus (Reny 2021).

The government also imposed administrative hurdles on the INGOs. Visa restrictions have been an issue for the INGO as many experts from other countries were called to support the response efforts, but the government allowed exceptional visas only for search and rescue teams and not for all aid workers.^14^ When the INGO advocated for an easing of the visa limitations, the reply from the governmental side was to “hire Turkish people instead”.^15^ These regulatory measures are indicative of a broader strategy employed by autocratic regimes, wherein they choose to regulate humanitarian organizations rather than outright suppress them, allowing their existence while imposing administrative obstacles. The allure of potential political advantages leads them to favor bureaucratic hurdles over repression.

Hiring Turkish nationals has been pushed as a requirement by the Turkish government after the 2017 foreign NGOs purge (Mellen & Lynch 2017). Most emergency responders were Turkish. One team member recalls how the coordination meetings were usually held in Turkish, excluding internationals from taking a meaningful part in the coordination:

“*In the coordination meetings, actually the majority of the organizations there are local organizations. I think they kept very…they tried to secure the local nature, the local identity if I can say, of coordination mechanism here. So, many of these structures hold meetings that take place in Turkish*”*.*^16^

When the earthquake struck, one of the INGO’s country office was destroyed and operations had to be relocated to a tent. For financial reasons, they had to downsize their workforce. However, due to the need to maintain the national–international staff ratio, they were unable to let go of the cleaning staff. This resulted in an absurd situation where the INGO had to lay off personnel directly involved in the response efforts, while the cleaning staff lingered in the tent all day making coffee.^17^

The foreign NGO purge by the Turkish government in 2017 has been a traumatic event for many INGOs which has had implications on their relationship with the government even after the earthquake. The INGO feared investigations and the possibility of being expelled for minor irregularities. They avoided any action that could be read as an interference in the government’s leadership. For example, one of the expats’ roles had to be changed in official documents as it included the words “camp management,” which might be perceived as an interference with AFAD’s control over formal camps.^18^ Because they feared that the government may be suspicious of information collection over smartphones, the organization collected beneficiaries’ data on paper, adding additional work.^19^

Due to state control over the response and the risk of imprisonment for dissent, it is unsurprising that the reports from state or state-controlled organizations express no negative critiques of the response. In an unexpected public criticism, the shelter sector expressed concerns about the excessive government control over the response. They state that government mistrust hindered clarity on response options for international organizations, alongside limited humanitarian access to certain locations. Moreover, the government limited access to data of damage assessments and response efforts, and other data from authorities had many discrepancies and missing data (Shelter Cluster 2023).

An assessment of the overall earthquake response also concluded that there was a lack of publicly accessible data on humanitarian needs (ACAPS 2023). This suppression of information enabled the government to maintain control and hindered INGOs from taking the lead in the response. Furthermore, it prevented INGOs from conducting needs-based assessments, hindering their ability to review aid distribution solely based on requirements. Consequently, the government could better serve its political base without facing direct allegations of biased aid distribution, as evidence of favoritism was not apparent.

This section illustrated how the government, primarily through AFAD and the TRC, asserted control over the humanitarian sphere. This nationalization was driven by the government’s political objectives, particularly in light of impending elections. Erdogan sought to convey the image of government dominance in the response effort, leading to the imposition of numerous bureaucratic obstacles for international organizations. The INGOs were coerced into a facilitative role, compelled to endorse the state agenda and support the effectiveness of the regime; lastly, they face expulsion or other administrative constraints.

### Access for INGOs Through Finances

In the humanitarian sector, financing is based on a top-down approach, with international organizations sub-granting projects to local NGOs. This setup tends to create a power imbalance that predominantly favors INGOs as the primary decision-makers (Khan & Kontinen 2022). In this way, partnership perpetuates unequal relations by fostering subordination (Contu & Girei 2014). This was evident in the composition of the emergency team, where all senior roles were filled by expatriates from the INGO.

The majority of the interviewees agreed that whoever holds the power in the relationship between agencies is the organization that has access to and availability of funding. INGOs have sub-grant agreements with the national NGOs who implement projects while INGOs take the decisions.^20^ Theoretically, local organizations can participate in coordinating the response through money from international organizations, but they still have a subordinate role in these meetings. Although the coordination meetings were held in Turkish, INGOs still tried to have some control over the conversations in these meetings. One interviewee felt that national NGOs had to be also careful in not antagonizing INGOs:

“*But if you [the INGO] and your local partner are in the same meeting, and you're saying something stupid they cannot talk back to you because you're the one who's giving them money*”*.*^21^

The emergency team justified this distinction through their view of a less formalized structure among national NGOs, which operate with fewer regulations and standards when providing services.^22^ In their perspective, INGOs are seen as more professionally oriented, adhering to best practices and global standards.^23^

The contrast between local and international actors aligns with Roepstorff’s (2020) concept of framing the local in opposition to the international, which has the potential to perpetuate stereotypes and power imbalances. This dichotomy underscores the idea that INGOs exhibit a more pronounced commitment to humanitarian principles, while national NGOs are community-focused and may be considered partisan in the sense that they primarily serve their represented community and may be easily involved into corruption scandals.^24^

The situation is intriguing. The government, led by organizations like AFAD and co-opted entities like TRC, dominates the humanitarian space, perceiving international organizations as mere funders without control. However, staff members within INGOs believe they influence local NGOs through their finances. This seems like a discrepancy in perception of who is controlling whom. The engagement of the INGO with the public authorities was described by one of the interviewees as follows:

“*[Public authorities] have very clear and high expectations of what we're able to provide, and there's clearly an expectation of direct budgetary support, or direct handover of materials that is not really in line with emergency humanitarian practice.*”^25^

This perspective may suggest a certain frustration on the part of the speaker, indicating that they perceive the government as unrealistic in their demands. The phrase “not really in line with emergency humanitarian practice” positions the speaker as an authority on proper humanitarian protocols. It reflects an underlying belief that their expertise should guide the response, suggesting a lack of trust or respect for the government’s decision-making processes.

In fact, the government had full control over both national and INGOs in their operations. Additionally, INGOs had to collaborate with co-opted or government-aligned national organizations to gain access. Nonetheless, INGOs had some influence over national NGOs through their significant financial resources. While they cannot control programs, they maintain access. Erdogan likely aimed to completely nationalize the earthquake response, but realized the need for a swift and efficient response in light of the elections. This required the financial support of international actors. This interdependence is visually depicted at the outset of the analysis in Fig. 1.

### Discriminatory Response

The effect of this nationalized earthquake response was particularly devastating for the Syrian refugees who were discriminated against in aid distributions. The government’s political strategy had already turned the refugees into a core issue of the election prior to the earthquake, with the clear intention of deporting the majority of refugees back to Syria (Syria Justice and Accountability Center, 2023). The AFAD settlements remained inaccessible to both Syrian refugees and INGOs (Hölzl 2023). INGOs had to assume the role of service providers and formalize agreements with AFAD to deliver services within the camps, while direct communication with the settlement’s residents was prohibited. Simultaneously, it was evident that assistance within these settlements was exclusively provided to Turkish citizens. One emergency team member explained how the organization tried to avoid working in the camps that were for Turkish citizens only:

“*There are many reports that have to do with discrimination in access to aid, which means that Syrian people are usually denied access to formal camps where more services are available. […] This is a very clear boundary for us that we don't want to go there because then we question neutrality*”*.*^26^

Further aid discrimination allegations came out. For example, during the response, state organizations’ definition of vulnerable populations was limited to vulnerable Turks (Türk Kizilay 2023). In some makeshift survival tents, Syrian families were kicked out to make room for Turkish families (Dadouch & Loveluck 2023). Moreover, the government imposed that at least 50% of the beneficiaries of the earthquake response needed to be Turkish citizens even though Syrian refugees were more vulnerable and more negatively affected by the earthquake. Municipalities identified beneficiaries of aid and then referred the names to the INGOs. The INGO staff was stopped at police checkpoints and questioned whether the organization was helping only Syrians or also Turkish people.^27^

On the operational level, the targeting of aid recipients became a minefield. The first beneficiaries contacted had already received aid before the earthquake, so they were mainly (if not only) Syrians. Furthermore, INGOs were restricted from implementing outreach activities in informal camps which hampered the capacity of INGOs to identify needs and deliver protection services.^28^ The lack of access to assess the needs of the refugees is also reported in several OCHA reports. Official figures of refugees are based on local government sources rather than assessed directly by the agencies.^29^ Finding Turkish beneficiaries and reaching the 50% ratio proved to be challenging for the INGO because the Turkish social security apparatus also delivered economic compensation to them. Hence, the 50–50 imposition from the Turkish government forced the INGO to operate outside their primary mandate as a condition to deliver aid.^30^

“*Our mandate here is to help the Syrian refugees, but all of a sudden we are now trying to help Turkish citizens. So also I think there is a struggle within the organization to maintain that neutrality*”^31^

The organization associated with the emergency team had prior involvement in the Syrian refugee response before the earthquake. This experience may have contributed to a negative bias towards the Turkish government among some team members. Their earlier interactions could have shaped perceptions of the government’s policies and actions regarding refugee support. Additionally, the government’s directive to prioritize assistance for Turkish citizens may have further exacerbated any existing biases. This demand leads to frustration and skepticism about the government’s commitment to humanitarian principles. Such sentiments are important to consider when interpreting the insights gathered from the team, as they may influence their perspectives on the current humanitarian response.

The nationalistic sentiment was also strategically used by the government aiming to restore its reputation among the Turkish population as the government has been heavily criticized for the slow response to the earthquake (Hubbard 2023; Reuters 2023). The discrimination between Turkish and Syrian IDPs reflects an anti-immigrant nationalistic discourse that was popular before the earthquake and was easy to pivot in the aftermath of the tragedy (Dhingra 2023). The proportion of Turkish citizens demanding that refugees be returned to their home countries jumped from 49 to 82% between 2017 and 2021 (Hattam 2023). Articles and reports from advocacy groups shared episodes of discrimination and scapegoating towards Syrian refugees in the aftermath of the quakes, to the point that some Syrians were scared to ask for help in Arabic (Hölzl 2023). Syrians were accused of lootings by Turkish communities and reports of verbal and physical abuse also by the authorities towards the refugee communities increased the overall tensions between the two groups (Human Rights Watch, 2023; Medina 2023).

For example, the region’s textile industry is a prominent export sector. Over half of its garment workforce, predominantly Syrian refugees, operates informally. Major western clothing brands are relocating production from Asia to Türkiye. They granted no deadline extensions after the earthquake, leading to increased exploitation of illegal migrants by production firms (Göçer 2024). With unemployment of migrants surging from 10 to 40% post-disaster, refugees are more susceptible to exploitation (Türk Kizilay 2023). The government tacitly supports this, benefiting the overall Turkish economy—a pivotal factor in Erdogan’s electoral strategy, especially as illegal migrants lack voting rights (Cohen 2024).

The distinction between Turkish and Syrian beneficiaries was central. The 50/50 ratio agreement fundamentally compromised impartiality, which requires providing aid solely based on need, and neutrality as the imposition came from the authorities. To enter the humanitarian space, the INGO had to comply with the government’s political calculations, compromising the implementation of neutrality and impartiality.

While the politicization of aid exists in both electoral and non-electoral autocracies, the mechanisms and motivations behind this process differ. In non-electoral autocracies, such as China or Myanmar, the absence of electoral competition allows regimes to focus primarily on maintaining the loyalty of elite coalitions and ensuring control over civil society organizations (Heurlin 2010). In such contexts, humanitarian crises provide an opportunity for the regime to consolidate its power by restricting international actors and leveraging aid distribution to reinforce patronage networks (Wood & Wright 2016). In electoral autocracies like Türkiye, however, the presence of elections introduces an additional layer of complexity. Here, regimes must not only maintain elite coalitions but also appeal to the general electorate, often using aid distribution as a way to showcase the government’s competence and responsiveness (Chang & Berdiev 2015). The upcoming presidential elections in Türkiye in 2023 heightened these pressures, pushing the government to centralize control over the earthquake response in order to claim credit for the aid delivered, a strategy that mirrors those used by other electoral autocrats facing crises (Windsor et al. 2014).

## Conclusion

The Turkish government shaped the humanitarian space by co-opting national organizations and limiting access of INGOs through national NGOs. In practice, this led to a situation in which the state exerted control over both INGOs and national NGOs. National NGOs also influenced INGOs through directives from the State, while INGOs had some leverage over national NGOs by virtue of their role as financial donors. However, both INGOs and national NGOs lacked the capacity to exert influence over the State and could not depoliticize the humanitarian space. This national humanitarianism was driven by government interests rather than the needs and voices of the affected population. The government’s aid allocation funnel discriminated against minorities without voting rights, particularly Syrian refugees. The timing of the earthquake, occurring just a few months before the presidential elections, made this discrimination especially evident.

Türkiye’s 2023 earthquake response shows the distinct challenges that electoral autocracies face when managing large-scale humanitarian crises. The proximity of the earthquake to the presidential elections added a layer of urgency for Erdogan’s regime, which was grappling with declining public support due to economic mismanagement and rising inflation. This electoral pressure incentivized the government to take full control of the aid distribution process, using it as a tool to project competence and to marginalize opposition strongholds. The nationalization of the humanitarian response—where INGOs were required to operate through government-affiliated NGOs such as the TRC—demonstrates how the Turkish government sought to claim credit for the aid provided while minimizing the visibility of international actors. This strategy is in line with theories of authoritarian resilience, where electoral autocrats must continuously balance the need for international legitimacy with domestic control over aid distribution (Gerschewski 2013; Paik 2011).

In summary, autocratic governments politicize natural disasters, as illustrated by the 2023 Türkiye earthquake. This study highlights the tensions between political expediency and humanitarian principles, demonstrating how authoritarian regimes exploit crises to reinforce their hold on power, a concept well-documented in regime survival theories. Autocratic leaders often use crises to marginalize vulnerable populations and consolidate control, manipulating humanitarian actors to serve political ends. The findings emphasize the importance of comprehensive disaster management frameworks that prioritize impartiality, inclusiveness, and accountability. Such frameworks are crucial in environments where authoritarian regimes seek to manipulate crises for political gain, ensuring that aid reaches all affected populations fairly and without discrimination.

The findings from this study align with the literature on autocratic governance, particularly regarding the co-optation of civil society. Authoritarian regimes often co-opt or suppress civil society organizations to prevent them from challenging state authority, using crises as opportunities to further entrench their power. Future research in this area could delve deeper into the long-term sociopolitical implications of nationalized disaster relief under autocratic rule. Furthermore, comparative studies across different authoritarian regimes and disaster types could provide insights into generalizable patterns and variations in crisis management strategies, contributing to the broader literature on regime survival and autocratic governance. Examining the role of civil society actors, including grassroots movements and interest groups, in mitigating the negative impacts of politicized humanitarian actions could offer valuable perspectives for building resilience and fostering democratization amid crises. Understanding the connection between authoritarian governance and humanitarian action is crucial for advancing both theoretical understanding and practical approaches to promoting human security and rights during times of crisis.

## Footnotes

1 We analyzed more than 100 situation reports. For a more detailed description of the reports, see Appendix.

2 The INGO requested to remain anonymous. Whenever we talk about “the INGO,” we mean this organization.

3 Interview 3; 4; 5; 6.

4 Interview 1.

5 Interview 2.

6 https://fts.unocha.org/appeals/1150/recipient-types

7 https://response.reliefweb.int/turkiye/sectors-activities-who-doing-what-where

8 Observation by the author.

9 Interview 1.

10 Interview 5.

11 Interview 4.

12 Interview 6.

13 Interview 4 and 6.

14 Interview 3; Observation by the author.

15 Observation by the author.

16 Interview 4.

17 Observation by the author.

18 Observation by the author.

19 Observation by the author.

20 Interviews 1-6.

21 Interview 2.

22 Interview 5.

23 Interview 3; 5; 6.

24 Interview 5; 6.

25 Interview 6.

26 Interview 4.

27 Observation by the author.

28 Interview 4.

29 OCHA humanitarian snapshots Kharanmaras, Malatya, Hatay, Adiyaman as of June 8, 2023.

30 Interview 2.

31 Interview 2.
